# Data-driven multi-objective optimization for electric vehicle charging infrastructure

**DOI:** 10.1016/j.isci.2023.107737

**Published:** 2023-08-31

**Authors:** Farzaneh Farhadi, Shixiao Wang, Roberto Palacin, Phil Blythe

**Affiliations:** 1 School of Engineering, Newcastle University, Stephenson Building, Newcastle upon Tyne NE1 7RU, UK; 2 School of Computing, Newcastle University, Urban Sciences Building, Newcastle upon Tyne NE4 5TG, UK

**Keywords:** Electrical engineering, Energy engineering, Energy Resources

## Abstract

This paper presents a data-driven methodology combining simulation and multi-objective optimization to efficiently implement transportation policy commitments, using as a case study the electric vehicle (EV) charging infrastructure in Newcastle upon Tyne, United Kingdom. The methodology leverages a baseline simulation model developed by our industry partner, Arup Group Limited, to estimate EV demand and quantities from 2020 to 2050. Four future energy scenarios are considered, and a multi-objective optimization approach is employed to determine the optimal types, locations, and quantities of charging points, along with the corresponding total capital and operational expenditures and charging point operating hours. Quantitatively, the variations of the portions of different types of charging points for the four scenarios are relatively small and within 3% range of the total number of charging points. The optimal solutions put priority on the slower charging points, with faster charging points having smaller portions each around 10%–13%.

## Highlights

• Data-driven optimization method for electric vehicle charging infrastructure planning
• Combining simulation and multi-objective optimization for future EV charging stations
• Cost reduction with higher diversity in the charging types and higher power points

## Graphical abstract

**Figure:**
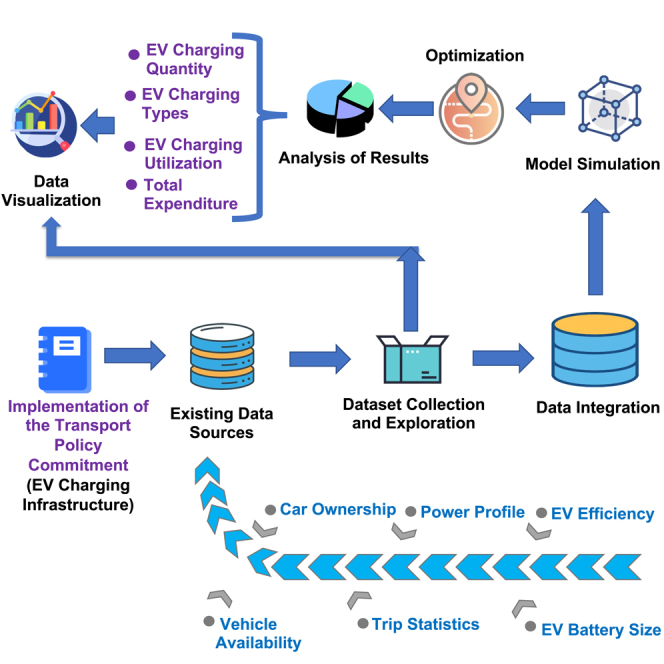

## Introduction

With the advances in science and technology to better understand and evidence the effects of climate change, individuals and governmental organizations are paying more attention to alternative forms of energy obtained from solar, wind, hydroelectric, and geothermal power.^1^ Many countries are progressing in the path of introducing appropriate policies to reduce carbon emissions and mitigate the effects of climate change. Most notably, the 2021 United Nations Climate Change Conference (COP26) which was held in Glasgow, Scotland, hosted delegates from 200 countries. The outcome of COP26 was a new deal, known as the Glasgow Climate Pact. The UK already committed in 2019 to a legally binding net zero target by 2050 and introduced new interim targets to reduce emissions by 78% by 2035.^2^

The Department for Business, Energy, and Industrial Strategy (BEIS) of the UK government has proposed a “net zero Strategy” to support the successful implementation of the strategy by investing over £100 billion in efforts to eliminate carbon emissions.^3^ As a key tenet of the new technologies to reduce carbon emissions, electric vehicles (EVs) are making a rapid sales progress with a yearly sales increase of 20% in 2022.^4^ The UK plans to achieve 100% zero emissions on all new cars and trains by 2035 by actively pursuing the development and sale of ultralow emission vehicles and zero-emission vehicles. To address the inevitable increasing demand for charging EVs, the BEIS has committed to a minimum of 2,500 charging points across the strategic road network in the UK.^5^

With the rapid increase in the variety and number of EVs, it is becoming challenging to meet the growing demand for charging these EVs. The challenges include the electricity grid overloading, forecasting the required charging load, and charging time and traffic-crowd management at charging stations.^6^ The research conducted by Illmann and Kluge^7^ studies the relation between an increasing availability of public charging infrastructure and consumer decisions to switch to EVs. They find evidence of a positive long-run relationship, and conclude that consumers also attach more importance to the charging speed. Therefore, governments and experts are interested in satisfying the demand for EV charging and maximizing the economy and benefits. Cai et al.,^8^ Yang et al.,^9^ and Wang et al.^10^ have studied and simulated the optimal location of charging stations for taxis and buses and have extended this to the location of charging stations for private EVs. Although the expectations and constraints for taxi and bus charging are very different from that for private vehicles, these studies highlight the importance of developing simulation models for EV charging infrastructure, which is also taken as the core contribution of this paper. On the other hand, LaMonaca and Ryan^11^ provide a review of the EV charging infrastructure market, and identify the relationship between governments, investors, and individuals by presenting and analyzing its essential functions and the roles of the players that will fuel the deployment of large-scale EV infrastructure. They find that assigning clear roles to the public and private actors and funders is needed to achieve efficient development of the required infrastructure for large-scale EVs.

Many studies look at the market, the economy, and the environment as entry points for designing the location of charging stations. However, there are still some deficiencies in the study of the actual quantity and types of charging points. There is a lack of appropriate analysis of the problem at the micro level (i.e., the spatial distribution of the charging points in a geographical region). This may lead to an inappropriate utilization of resources and an unnecessary burden on the power grid: having an unbalanced charging demand in different areas of the region may violate the constraints of the existing electricity grid distribution. Satisfying these constraints is detrimental to the development and implementation of policy commitments on the new EVs in the long run. Therefore, it is necessary to comprehensively analyze and optimize the type, quantity, location, and total capital and operational expenditures (CapEx and OpEx) of charging points from a combination of micro and macro levels.

In order to help city councils and other governmental organizations plan for the EV charging infrastructure, this paper proposes an optimization method based on *genetic algorithm* (GA) modified by deep learning and neural network architectures. The goal of the optimization is to consider and optimize the following factors needed to design and expand the EV charging infrastructure: charging point type, charging point location, charging point quantity, total capital and operational expenditures, and operating hours of charging points.

The open-access statistics published by the official authorities are used in the modeling and optimization developed in this paper (cf. the next section and the references therein for the datasets used in the case study). The geographic and vehicle data for 175 districts in Newcastle upon Tyne are obtained. Newcastle is the largest city in the north east of England. The districts are called lower layer super output areas (LSOAs).^12^ These LSOAs are geographical divisions that are small enough for capturing essential details in an accurate simulation model, and at the same time large enough for reducing computational complexity of the developed simulation model. These datasets are then analyzed using statistical methods and optimized using the designed optimization model. The results are then further evaluated by providing a sensitivity analysis of the results and comparing with traditional GAs.

Previous studies generally rely on restrictive assumptions that limit the scope for application of the results. Studies that provide optimization for a single type of charging point are available.^13^^,^^14^ In order to better investigate multiple aspects of EV infrastructure planning at the same time, this research uses GA, which is improved based on the concepts of recurrent neural networks (RNNs) and fuzzy logic. The main contributions of this paper are as follows.

1. This study integrates simulation and optimization for EV charging infrastructure planning of a city in the UK. This integration allows for informed decision-making by considering factors such as charging point types, locations, quantities, total capital and operational expenditures, and operating hours.
2. The study develops a comprehensive data-driven methodology that integrates a multi-objective optimization approach with a baseline simulation model, capturing the EV demand and quantities for the period from 2020 to 2050.
3. The optimization results provide insights into the optimal configurations of charging infrastructure, considering the trade-offs between meeting charging demand and minimizing costs. The study identifies the spatial distribution of charging points, highlighting areas with high demand and recommending appropriate charging point types and quantities.
4. By drawing on RNN architectures, word embedding models, and long short-term memory (LSTM) networks from machine learning literature, the traditional GA is extended and combined with fuzzy logic to design a multi-purpose decision model for multi-objective optimization problems.
5. The designed model removes the need to compress multiple objective functions into a single objective function. Instead, the underlying simulation environment is modeled using actual data, enabling a transition from function-driven to data-driven optimization and evaluation.
6. An implementation of the computations are provided using vector and matrix representations. Matrices give a compact way of handling large volumes of data and updating values efficiently.

### Related work

Researchers have recently used traditional GAs for performing optimizations to find effective solutions for problems of the EV charging infrastructure. Efthymiou et al.^15^ have studied the use of GA for designing the location of EV charging stations in Thessaloniki, Greece, using the origin-destination data of conventional vehicles. This study is limited to the required charging stations in 2020, with the results indicating that 15 stations would be required to cover 80% of the estimated EV charging demand. Jordán et al.^16^ have studied the use of a multi-agent based simulator of urban fleets, called SimFleet, for optimizing the location of EV charging stations in Valencia, Spain. Combining GA with graph theory is explored in the work by Altundogan et al.^17^ to find the optimal location of charging stations. The work by He et al.^18^ provides a joint optimization of electric bus charging infrastructure, vehicle scheduling, and charging management for the Salt Lake City, UT. The problem is formulated as a mixed-integer non-linear optimization and is solved via a GA-based approach. Machine learning methods for estimating and predicting the effect of transport policy interventions is studied recently using case studies from clean air zone^19^^,^^20^ and adoption of EVs.^21^

Optimal planning of public charging infrastructure for joint transportation and distribution networks is reviewed in the work by Unterluggauer et al.^22^ The use of machine learning algorithms for optimal planning of EV charging stations is reviewed by Panda et al.^23^ A bilevel heuristic optimization approach is proposed by Li et al.^24^ for selecting the charging stations and performing route planning of EVs in a large city in China. he work by Abdullah-Al-Nahid et al.^25^ has focused on the interaction of the vehicle with the power grid and provides an EV charging scheme for centralized residential charging stations considering both consumer satisfaction and power network stress. The work by Mirheli and Hajibabai^26^ proposes a hierarchical optimization in a game framework for an integrated design of the charging infrastructure and utilization management of the charging facilities while accounting for charging agency and user perspectives.

Ge et al.^27^ provides a method to compute the optimal location of the charging stations by taking into account the traffic density and other restrictions. The work has proposed a partitioning method to divide the planning area into square cells and employed a GA to select the best location of the EV charging stations by randomly generating the traffic flow within the cells. Zhou et al.^28^ have studied optimal locations for the EV charging infrastructure in Ireland by building a comprehensive social cost model. The work considered the Irish land area as a rectangle of size 350×200 sq. km and partitioned it into smaller cells. The developed model iteratively generates the best arrangement of charging locations by placing charging stations at the intersection of cells, considering five significant cities as transport hubs, and developing radial divisions around the hubs. Ren et al.^29^ have developed a gray decision making model to optimize the location of EV charging points. The model integrates partially clear and unclear information with uncertainty, and utilizes genetic algorithms to optimize the location of charging points in selected areas of Nanjing, China. Cui et al.^30^ have considered finding the most suitable location for building EV charging facilities as a multi-criteria decision-making problem. The problem is then assumed to be a group decision–making task that has contradictory criteria with compromising being adequate for conflict resolution. In this work, fuzzy set theory is used to deal with inaccurate data in the model. The advantages of the proposed EV charging station model is shown on a practical example from Shanghai, China.

The studies mentioned above generally make limiting assumptions, focus on the power distribution aspects of the problem, require precise models, consider fleets of EVs, formulate single objective optimizations, or consider only a short planning horizon. In contrast, we develop a data-driven multi-objective optimization for the EV charging infrastructure planning over a long time horizon.

The rest of this paper is organized as follows. First, the data-driven simulation and optimization framework for transition toward EVs is presented. This includes the EV charging simulation model and the novel multi-objective genetic algorithm. Second, the results of applying the developed framework on the EV charging infrastructure planning of a city in the United Kingdom. Third, sensitivity analysis of the results and comparison with traditional genetic algorithms are provided. Finally, concluding remarks are provided.

### A data-driven simulation and optimization framework for transition toward electric vehicles

Greenhouse gas emissions are considered to be one of the main causes of the recent increase in extreme weather events (e.g., more frequent flooding, extreme rainfall, and longer periods of draught). According to United Nations’ Climate Action, more than 70 countries have set a net-zero target, covering 76% of global emissions. The UK government has proposed “net zero strategies”.^3^ Whilst there are a range of ways in which net zero could be achieved, the UK set out a delivery pathway showing indicative emissions reductions across sectors.^31^ For the research of this paper, the transport sector is considered. The Department for Transport^32^ provides detailed information on key policies in the transport sector of net zero emission strategy: increasing cycling and walking; zero emission buses and coaches; decarbonizing railways; a zero-emission fleet of cars, vans, motorcycles, and scooters; accelerating maritime decarbonization; and accelerating aviation decarbonization.

The research of this paper is focused on one of the net zero policies, which is *a zero-emission fleet of cars, vans, motorcycles, and scooters*. For delivering the policy, one of the most important commitments that requires advance planning, implementation, and methodology is *to ensure the UK charging infrastructure network meets the demands of its users*. This paper proposes a data-driven methodology for the implementation of this policy commitment by expanding the current EV charging infrastructure. This section describes the EV charging infrastructure problem setting in more details with focus on Newcastle upon Tyne, UK, and formulates an optimization method for finding the best location of charging stations.

#### Framework for optimal implementation of policy commitments

**Figure 1:**
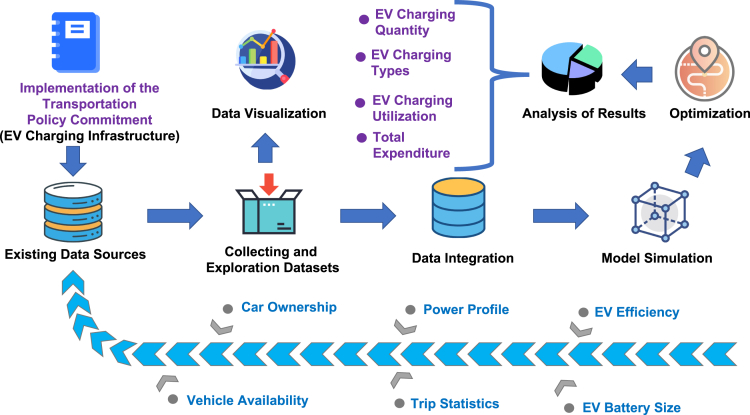
Framework for the net zero emission policy commitment of providing EV charging infrastructure The framework shows the process of gathering the suitable datasets relevant to the policy commitment and build a model that can simulate future scenarios for optimization purposes to achieve a better implementation of policy commitments in transport systems.

A framework for finding the best implementation of policy commitments in transport systems has been designed. According to the Figure 1, a key policy in the transport sector of net zero emission strategy has been selected as a case study. The policy will be achieved through various commitments including incentives for individuals and companies to buy EVs, and providing the required EV charging infrastructure. In order to achieve these commitments, the required number of EV charging points and stations will be increased in on-street and public places to create a better availability and accessibility of the charging points for the EVs.

A model is built in the research of this paper for simulating the net zero emission policy commitment on EV charging infrastructure to compute the increase in the quantity of charging points with different types. The model includes two distinctive stages of simulation and optimization. The simulation stage of the baseline model has been taken from the industrial partner of this project, Arup Group Limited.^33^ The assumptions used for the construction of this baseline simulation model have been selected and revised according to the available data. The relevant subsets of the output of the simulation model have been used for feeding the optimization stage of the model. The main contribution of the results presented in this paper is on the development of a novel optimization approach to find the optimal location of EV charging points (best implementation of the policy commitment) utilizing the baseline simulation model provided by Arup.

The scheme for building the simulation model and integrate it with optimization is presented in Figure 1. The scheme shows the process of gathering the suitable datasets relevant to the policy commitment and build a model that can simulate future scenarios for optimization purposes to achieve a better implementation of policy commitments in transport systems. The following steps are taken to build the scheme.

1. Selecting transport policy commitment from net zero emission strategy as a case study.
2. Analyzing the policy commitment of providing EV charging infrastructure by performing a suitable literature review in order to understand the objectives of the policy commitment.
3. Analyzing assumptions, datasets, and calculations used by Arup Group Limited in the construction of the baseline simulation model.
4. Analyzing future energy scenarios developed by National Grid and used for constructing the simulation model.
5. Computing the estimated power demand and EV quantities for the years 2020–2050 for four future energy scenarios.
6. Building an optimization approach using the simulation model for providing an efficient expansion of EV electrical infrastructure with respect to the charging point quantities, their types, locations, costs, and operating hours.

### Electric vehicle charging simulation

Charging points will serve users differently. In this study, two broad categories are considered for the location types to make a better connection with the trips estimated from road use statistics.

• **Off-street Location Type.** charging that is related to home charging.
• **Public Location Type.** charging that is an aggregation of destination, on-street residential charging, and on-route charging.

The chargers of EVs are categorized by the charger ratings (kW). The current popular charger ratings are summarized below.^34^ Note that the charging point technology might change in the future, therefore more charger ratings can be introduced to the EV charging infrastructure, and the below definitions and names might change.

• **Slow charging points**: These types are 3 kW chargers. The length of charging with these charging types usually takes around 6–12 h. This type of charger is mostly used for household purposes.
• **Fast charging points**: These types are 7 kW, 11 kW and 22 kW. It can take around 3-4 h to fully charge some models.
• **Rapid charging points**: These types are 50 kW. These chargers usually can charge to 80% in 30–50 min.
• **Ultra-rapid charging points**: are 100 kW and up to 150 kW. These chargers are added in order to charge quickly as possible within 20–30 min.

These types of charging points can be further classified based on the underlying charging power method that could be either *Alternating Current* (AC) or *Direct Current* (DC). The power that comes from the power grid is always AC. Electronic devices can have a converter built into the plug to store the power in the battery using DC.

In order to get a measure of priority for these types of charging points, six key factors for each of the charger ratings (kW) are considered. These factors include infrastructure and installation costs, utilization, impact on the grid, future proofing, and tariff.^35^ Table 1 shows the average prioritization computed as a weighted sum of these factors. A priority weight of 100% means that the charger is the most suitable option, whereas a priority weight of 0% indicates the charger is not as suitable.

**Table 1:** The priorities of different charging types computed by combining multiple factors

Charger Priority							
Chargers types	AC				DC		
	Slow	Fast			Rapid	Ultra-Rapid	
	3	7	11	22	50	100	150
Priority weights (%)	68	72	80	76	56	32	32

These factors include infrastructure and installation costs, utilization, impact on the grid, future proofing, and tariff.

#### Geographic area

**Figure 2:**
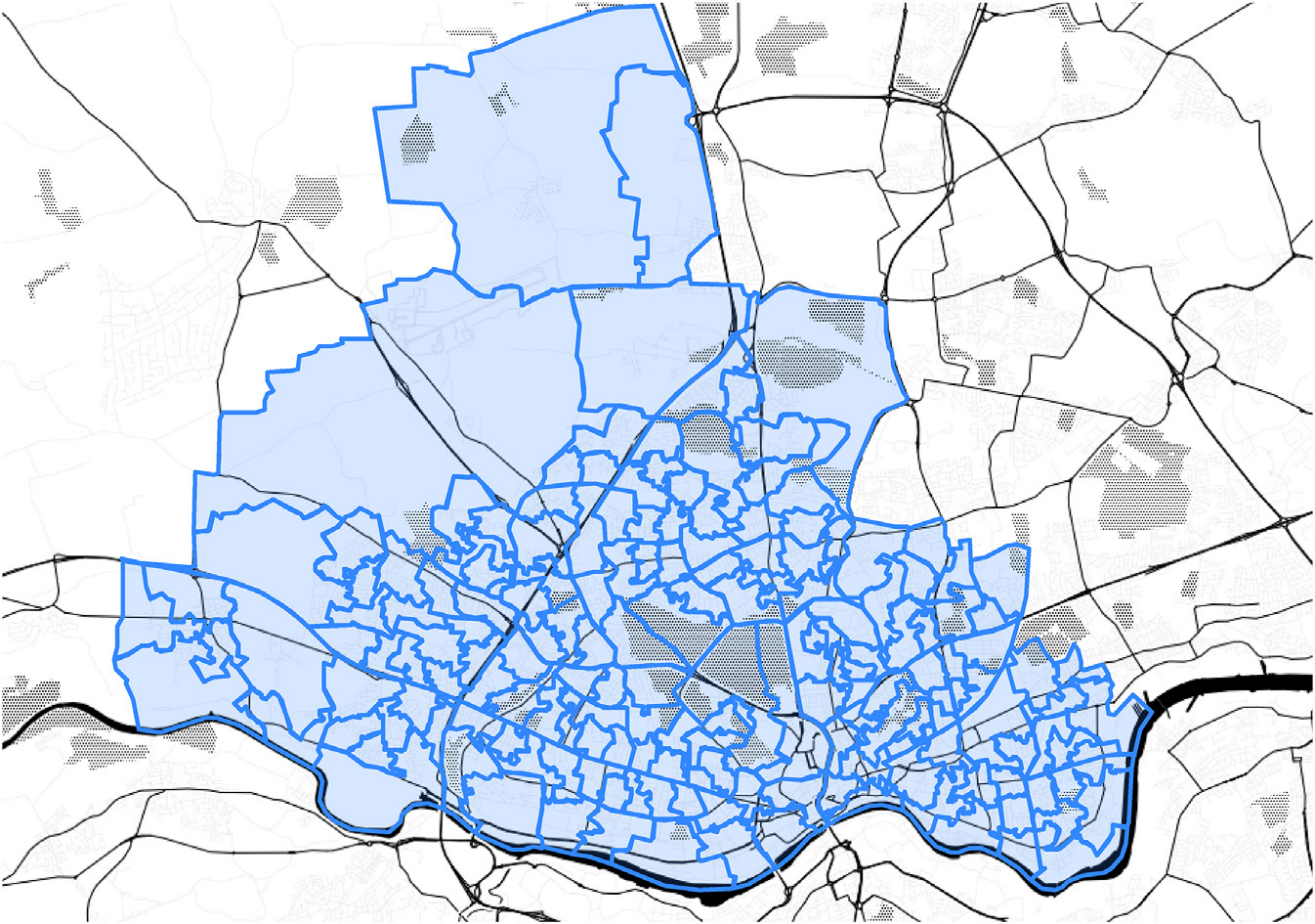
LSOAs of Newcastle upon Tyne There are 175 LSOAs in the map that will be used in this paper as geographical divisions.

There are three geographical divisions used for statistical purposes. These are called *Output Areas* (OAs), *Lower Layer Super Output Areas* (LSOAs), and *Middle Layer Super Output Areas* (MSOAs). The number of people and households in any of these areas is stated in Table 2. The OAs are the smallest division. The LSOAs have an average of 1,600 residents and 670 households. Currently, there are 34,753 LSOAs in England (32,844) and Wales (1,909). MSOAs have an average population of 7500 residents or 4000 households. In order to divide the geographical area of Newcastle upon Tyne to smaller unified regions, LSOAs are used to perform a refine analysis of the EV charging infrastructure. Figure 2 shows the LSOAs of Newcastle upon Tyne on its map. This map contains 175 LSOAs.

**Table 2:** Geographical divisions used for statistical purposes (Source: Office for National Statistics)

Area type	Lower threshold		Upper threshold	
	People	Households	People	Households
Output Areas	100	40	625	250
Lower Layer Super Output Areas (LSOAs)	1,000	400	3,000	1,200
Middle Layer Super Output Areas (MSOAs)	5,000	2,000	15,000	6,000

#### Datasets

To have a reliable prediction of the number of EVs and simulate a realistic environment, the datasets available from the government databases are used. The general approach taken is to predict the average vehicle mileage demand in each region and then obtain the EV charging demand using the datasets from the number of regional vehicle registrations and regional vehicle miles traveled. The trips are then considered to be either *home based* or *non-home based*. A home-based trip is a trip that starts and ends in the same LSOA. A non-home based trip is a trip that starts and ends in different LSOAs. A list of the datasets used in the baseline demand prediction is provided in the key resources table in the appendix.

#### Future energy scenarios

To plan and develop the transport infrastructure including EV charging points, it is essential to develop scenarios that clarify how users might engage and interact with the transport infrastructure and how the current set of policies affects this interaction at large scale. The future travel scenarios are constructed based on a range of factors that affect the future of the transport as elaborated in the rest of this section. It is important to emphasize that such scenarios are not predictions as there are large uncertainties around the user behaviors and technological developments, but they provide plausible futures. These scenarios are not intended to be good or bad statements, but are aimed at formulating the most plausible combinations of uncertain factors, and find possible actions that need to be taken or adapted to these scenarios as more certainties are revealed over time.

Despite the recent advances in the EV technology and the EV charging facilities, the number of EVs and the charging points are relatively low at the moment. As of March 2023, there are only 735,000 EVs on UK roads, which is less than 2% of the total licensed vehicles (according to report by United Nations Net Zero Coalition). This leads to large uncertainties in the future user charging behaviors. The requirements on the EV charging infrastructure depend on charging behaviors, the availability of the off-street parking, and mobility trends.

National Grid has considered four future energy scenarios to forecast the EV numbers. These four scenarios are based on the speed of decarbonization and the level of societal changes. The four scenarios are (1) **Steady Progression:** a pessimistic scenario with a slow speed of decarbonization of the energy vectors and low level of societal change, slow adoption of EVs and slow installation of charging points. The ban on new petrol/diesel vehicles is achieved in 2035 by cars and in 2040 by vans. (2) **System Transformation:** a scenario with a moderate speed of decarbonization and middle level of societal change. Charging points for EVs are installed ahead of the need. The ban on new petrol/diesel vehicles is achieved in 2032. (3) **Consumer Transformation:** a scenario with a moderate speed of decarbonization and higher level of societal change. Drivers adopt EVs ahead of charging provisions. The ban on new petrol/diesel vehicles is achieved in 2030. (4) **Leading The Way:** an optimistic scenario with a fast speed of decarbonization and highest level of societal change. The 2030 ban on new petrol/diesel vehicles is achieved.

Arup has developed four scenarios to forecast the number of EVs.^33^ These scenarios are adapted from the National Grid ones, and are as follows. **(1) Baseline:** A baseline set of assumptions that relies on the behavior of consumers to date to forecast EV energy demand and charging point quantities. **(2) Consumer Efficiency:** A scenario that assumes EV purchases are done mainly for every day short distance use. EV owners use more lower charger speeds and operate between 20% and 80% to optimize their battery life. **(3) Government On-Street:** Public residential chargers are made available through appropriate government schemes. **(4) Rapid Dominant:** Rapid and above charging points are made available to reduce consumer dwell times.

**Figure 3:**
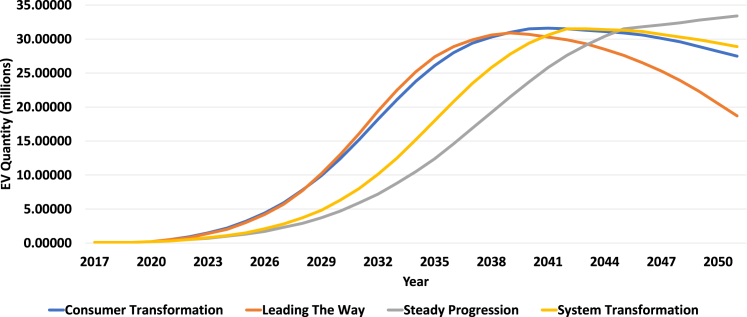
Estimation of the number of EVs for Newcastle upon Tyne These estimations are obtained using the future energy scenarios of national grid, which are also used in the scenarios developed by Arup.

Figure 3 shows the estimation of the number of EVs for Newcastle upon Tyne, which is obtained using the future energy scenarios of the National Grid proportioned to the number of cars Newcastle. These estimations are also used in the scenarios developed by Arup. Three of the four curves in Figure 3 show a reduction in the number of EVs on the road between 2042 and 2048. Steady Progression scenario shows a peak in 2050. The fall in EV numbers in the three scenarios meeting net zero by 2050 is due to National Grid’s assumptions surrounding other forms of propulsion, automated self-driving vehicles and public transport. According to the Figure 3 the peak year for Consumer Transformation is in year 2046, for Leading the Way is in year 2042, for Steady Progression is in year 2050 and for System Transformation is in year 2048. The peak year together with the estimated number of EVs are reported in Table 3.

**Table 3:** Peak year and the maximum number of estimated EVs until 2050 based on future energy scenarios of National Grid for Newcastle upon Tyne

Future Energy Scenarios	Peak Year	EV Quantity
Steady Progression	2050	160,403
System Transformation	2048	146,617
Consumer Transformation	2046	145,345
Leading The Way	2042	134,606

Next it is discussed how to estimate the number of EVs in each LSOA. Define the EV uptake percentage as the total number of forecasted EVs divided by the total registered vehicles from the National Trip End Model (NTEM). The NTEM also provides the *total registered vehicles* for each MSOA. This total registered vehicles for MSOA is split into the number of vehicles for LSOAs using a proportional split determined by the 2011 Census. The forecasted number of EVs in each LSOA is the number of registered vehicles in the LSOA multiplied by the EV uptake curve percentage:

(Equation 1) $${\text{EV}}_{\text{LSOA}}={\text{EV}}_{\text{uptake}}\times {\text{Veh}}_{\text{MSOA},\text{NTEM}}\times \frac{{\text{Veh}}_{\text{LSOA},\text{cen}}}{\sum_{\text{LSOA}}{\text{Veh}}_{\text{LSOA},\text{cen}}}$$

Note that this proportional split can be further refined by considering demographics and household income that may mean ownership in some LSOA will grow in different ways to others. The optimization methodology of this paper is general and can be adapted to take into account such factors. The next section will discuss the results of the optimization for Base Scenario from Arup in a combination of the estimated number of EVs for Newcastle upon Tyne from future energy scenarios in peak years for *public* locations.

#### Construction of the simulation model

**Figure 4:**
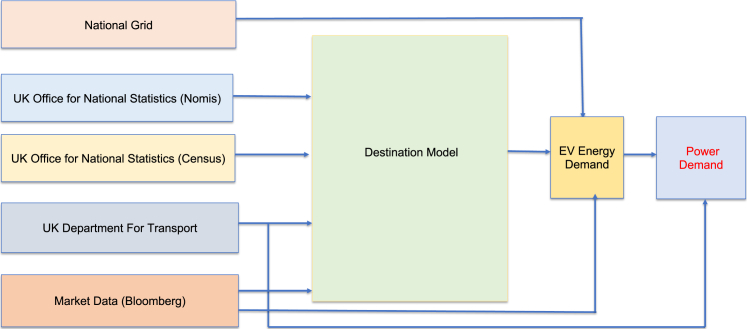
The flow chart of the proposed data-driven simulation for the EV charging infrastructure planning The simulation has a Destination Model that seamlessly integrates multiple data sources to estimate EV energy demand and power demand at a granular geographical level.

In order to simulate the EV charging infrastructure of Newcastle upon Tyne, the above datasets, the assumptions and calculations of this section have been used to estimate the total EV travel mileage, the energy demand, and the power demand. A summary of the calculations for estimating the average daily EV mileage is as follows. The national trip end model has been used for the Origin/Destination data. This dataset is open source and is provided by the Department for Transport. The dataset defines two types of origin: Home based and Non-home based. Each LSOA is assigned an urban-rural classification as defined by the Census data,^36^ for which the average annual mileage and the average number of car/van driver trips are used to define an average trip mileage for each of the four urban-rural classes. For all home-based trips, the average trip mileage for each rural and urban classification is applied. The non-home based trips is split into non-home based trips that start within the Local Authority area and non-home based trips that start outside of the given Local Authority area, using a census Local Authority origin destination dataset.^36^ For non-home based trips that start within the given Local Authority, the average trip mileage determined in the previous stage is applied. For non-home based trips that start in another Local Authority area, the traveled distance is calculated using spatial mapping. The EV travel mileage is computed by considering the total travel mileage, the trend in the growth of registered EVs and the total number of registered vehicles, as well as to the EV battery range. The general overview of the simulation has shown in Figure 4.

#### Optimization approach

This paper investigates multi-objective optimization methods in order to inform on the future EV charging infrastructure and find the best quantity of charging points, their types and locations whilst minimizing the total capital and operational expenditures. Based on the existing optimization methods, a new optimization solution is proposed in this paper to address the limitations of the standard single-objective optimization methods. More specifically, the optimization solution of this paper is different from previous ones in the following directions: (1) it is guided by LSTM models from the machine learning literature; (2) it does not compress the multiple objectives into a single objective, but combines and ranks multiple objectives through fuzzy logic; (3) it extends the capabilities of the traditional genetic algorithms by integrating available data with a simulation model.

#### Introduction to genetic algorithms

Genetic algorithms (GA) are popular global search and optimization methods that have made progress in the last two decades inspired by the natural evolution of organisms in nature, the natural law of survival of the fittest through selection and elimination, and random evolution and generation of offspring in order to obtain the best performing population in environmental adaptation. In recent years GA with elite strategies has been developed for multi-objective optimizations by Konak et al.,^3^ where elite samples are the class of populations that perform best compared to other genotypes.

In order to solve the problem of charging point locations and types, a multi-objective optimization decision problem is defined. In the past, multi-objective optimization problems have been solved by constructing a utility function that describes the relative importance of each objective. The problem is then translated to a single objective optimization. This requires substantial prior knowledge on the trade-offs and valuation of each individual objective. In contrast, the multi-objective nature of the problem is maintained and a novel optimization is developed that automatically encode the relations between the objectives in the solution process.

#### Comparison of GA to other optimization methods

There are other optimization methods inspired by nature and are developed for finding solutions of challenging problems. These optimization methods are generally called *Evolutionary Algorithms*. A survey of multi-objective evolutionary algorithms can be founds in the work by Zhou et al.^37^ A survey on the application of evolutionary algorithms in transportation is provided by Chen et al.^38^ Among these methods, Ant Colony Optimization (ACO) is a heuristic algorithm inspired by the foraging behavior of ant colonies.^39^ It is shown by Alexander and Sriwindono^40^ that while ACO is able to find better solutions than the GAs when the fitness function is known, the GA shows a better speed of completion. This feature is in particular important for the problem setting of this paper where there are multiple fitness functions and the optimization domain is very large. Therefore, this study has focused on adaptation of GA to the problem setting of this paper.

#### Optimization steps for EV charging

The proposed optimization approach does not compress multiple objectives into a single objective but combines and ranks multiple objectives through fuzzy logic. The optimization algorithm is guided by the long short-term memory (LSTM) models from machine learning literature. The optimization model of this paper is designed to adopt the most central idea of GAs, which is to simulate nature for selection, evolution and reproduction. By constructing a genotype complete with the main parameters possessed by a charging point, traits and expressions are designed to describe the characteristics of a charging point and how it will behave in a given environment. The main idea of the GA for solving the problem of finding the best location of charging points is as follows. The EV power demand generated from the EV users will be responded to by the EV chargers depending on the location of EVs and the charging points. The number of EVs that need to be charged will be different over time (different generations or iterations). The algorithm deploys different types of charging points at various locations to select the best locations and types. The details of the optimization steps can be found in the appendix. Next, we discuss how to apply the optimization for the EV charging infrastructure planning.

The following assumptions are made based on the processed data: (1) EVs will be charged according to EV charging demand, which means that users will charge according to their needs and will not necessarily wait for the EV battery to run out of charge completely; (2) users prefer more powerful charging points, which will allow them to have shorter waiting times for charging, but users will consider a combination of distance and time; (3) projected based on data, the initial number of EVs is selected according to Table 3. (4) EVs requiring charging are randomly generated within the LSOAs; (5) after comprehensive consideration, six types of charging points are selected for optimization: 7 kW, 11 kW, 22 kW, 50 kW, 100 kW, and 150 kW.

The steps for constructing the new optimization model are as follows.

1. Analysis and processing of data to determine the subject and environment of optimization.
2. Construct gene vectors, determine genotypes and score types, and construct the initial solution vector space, i.e., construct the initial population.
3. Through the processing of real data, a resource vector is designed through mapping and an environment matrix is constructed.
4. Study the specific expression of the design score type through the influencing factors of the real problem, and complete the construction of multiple objective functions.
5. Use the elite strategy of fuzzy logic to rate the score types.
6. Memorize and eliminate the initial solution vector matrix based on the ratings by constructing a transformation matrix. This is similar to the memory gate and forgetting gate of LSTM models.
7. Generate the next generation of children based on the rating content and child generation matrix, and add them to the solution vector matrix space.
8. The environment matrix is regenerated according to the mapping rules.
9. Rating by calculating the score type and then calculating the crowding by the inner product for the exemplar elite, so that the crowding is in the right zone to ensure species diversity and also the right direction for optimization and search.
10. Repeat steps 5 to 9 until the generational requirements are satisfied.

**Figure 5:**
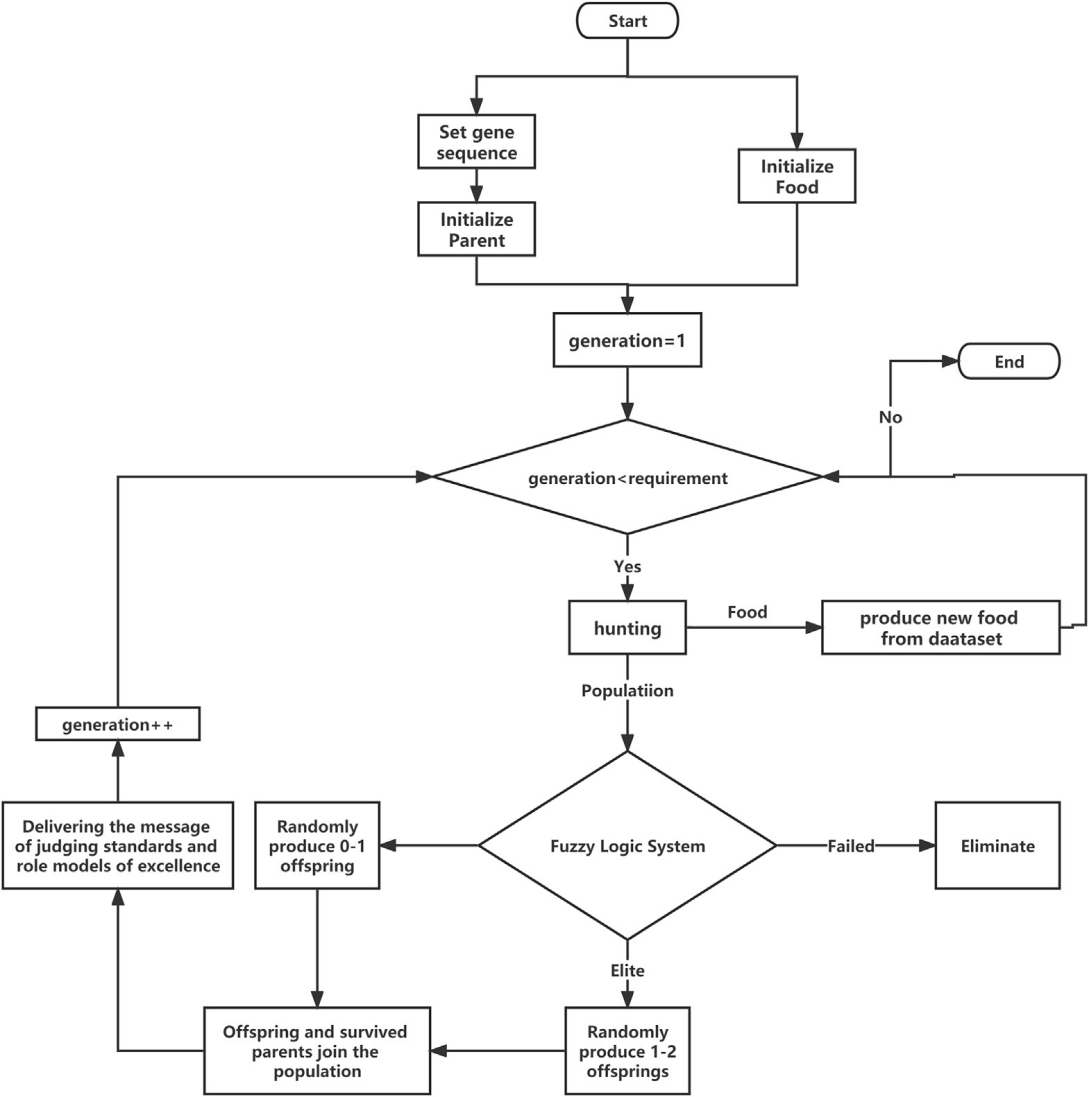
The flow chart of the new optimization based on Genetic Algorithm and Fuzzy Logic The steps construct a new optimization model by considering an initial population of gene vectors with genotypes and score types, and updating the population iteratively.

The steps are also shown in Figure 5 as a flow chart.

#### Constructing the solution vector spaces

Based on the optimization method proposed in the previous section, gene vectors are constructed for each EV charging point as

(Equation 2) $${\vec{X}}_{j}^{\left[i\right]}=\left(a_{1},a_{2},a_{3},a_{4},a_{5},b_{1},b_{2},b_{3}\right)\text{,}$$

where $a_{1}$ is the gene ID, $a_{2}$ represents the latitude, $a_{3}$ represents the longitude, $a_{4}$ is the charging type, $a_{5}$ is the charging point total capital and operational expenditures, $b_{1}$ represents the number of charges score, $b_{2}$ represents the charging point operating hours score, $b_{3}$ represents the generation of birth in the optimization algorithm. The relationship between these values will be discussed after introducing the environment matrix.

The total investment is computed according to the following equation:^28^^,^^41^

(Equation 3) $$a_{5}=C_{j}\frac{r_{0}{\left(1+r_{0}\right)}^{n_{year}}}{{\left(1+r_{0}\right)}^{n_{year}}-1}+M_{j}\text{,}$$

where $r_{0}$ is the inflation rate which is set to 10%, $C_{j}$ is the construction cost of each charging point that includes the acquisition cost, the land price, and the cost of replacement of the charging point at the end of its life span. $M_{j}$ is the maintenance costs and $n_{year}$ is the planned life span set to 15 years^42^

After designing the genetics of the charging point, the 175 LSOAs of Newcastle upon Tyne are considered with random selection of charging points of any type in the center of each LSOA. These gene vectors are then added to the space of solutions as in matrix ((0.2)) with m=175.

#### Generating the EV power demand

**Figure 6:**
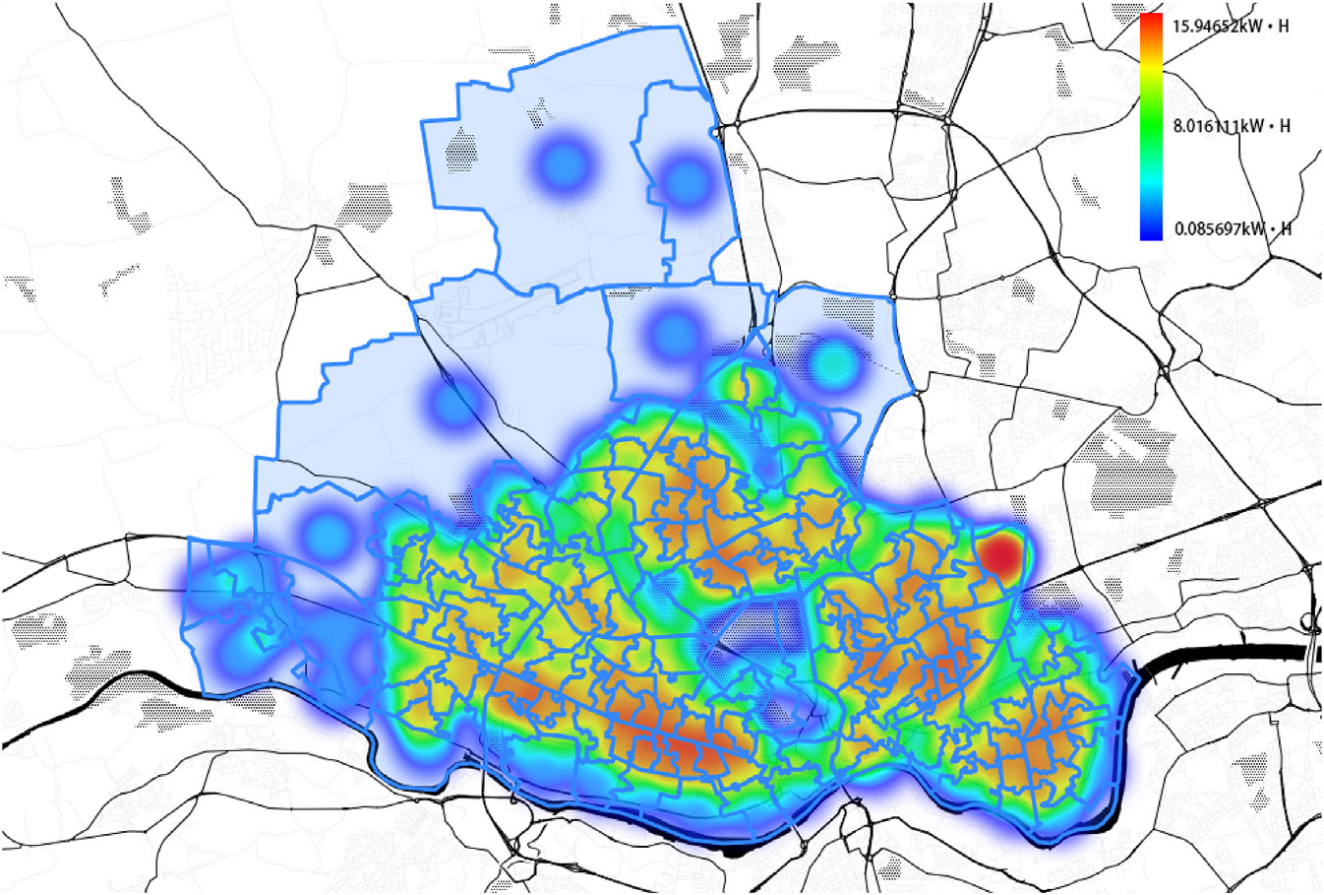
Heatmap of EV charging power demand in the peak year (2042) The visualization is performed for Leading The Way scenario.

**Figure 7:**
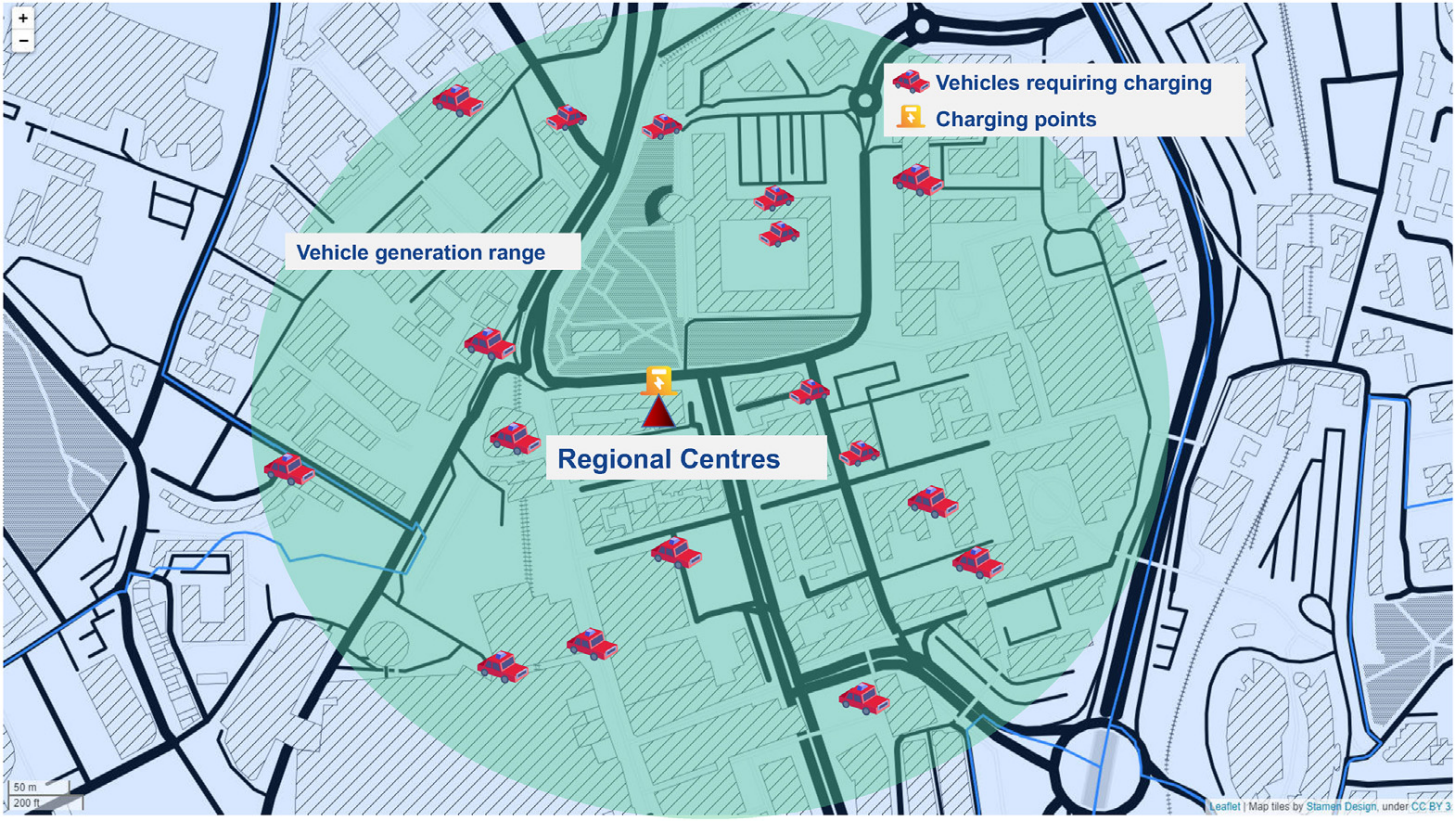
Initialization and EV charging power demand The visualization is performed for Leading The Way scenario.

A heatmap of the EV charging power demand is shown in Figure 6 for the peak year (2042) of the Leading The Way scenario. The initialization of EVs in one LOSA is presented in Figure 7.

The behavior of the EVs is simulated using the EV peak power demand as described in the previous section. The required number of EVs in each LSOA is generated randomly within a circle around the center points of the LOSA. Normal distribution is used to generate the location of EVs according to the following formulas

(Equation 4) $$\mu_{1},\mu_{2}∼N\left(0,1\right),A∼U\left(-\pi ,\pi \right)$$

$$\text{EV}\;\text{Latitude}=CL_{a}+\mu_{1}R\;\cos\left(A\right)$$

$$\text{EV}\;\text{Longitude}=CL_{o}+\mu_{2}R\;\sin\left(A\right)$$

$$R=\sqrt{\frac{1}{n}\sum_{i=1}^{n}\left[{\left(Ma_{n}-CL_{a}\right)}^{2}+{\left(Mo_{n}-CL_{o}\right)}^{2}\right]}\text{.}$$

The above formulas mean that the scaling factors $\mu_{1},\mu_{2}$ are selected randomly according to the normal distribution with zero mean and standard deviation equal to one N(0,1). The angle *A* is selected according to uniform distribution from the range [−π,π] to cover the whole circle. The latitude and longitude of the EV are then computed from the center point of the LSOA $\left(CL_{a},CL_{o}\right)$. These formulas ensure a normal distribution of EV numbers from the center to the edges of LSOAs.

The radius *R* is calculated as the average distance of the $\left(Ma_{n},Mo_{n}\right)$ to the center points. Here, $\left(Ma_{n},Mo_{n}\right)$ are the latitude and longitude of the $n^{\text{th}}$ marginal point. Since regional centers are mostly commercial centers or transport hubs, this means that EVs are likely to be there more often using normal distribution to simulate this behavior. EVs may engage in cross-region charging behavior for a number of reasons, e.g., when the charging points in other region are full.

The following equations are used to generate the total power demand, which is then used for creating the charging requirements of EVs.

(Equation 5) $$\begin{array}{l} \eta ∼N\left(0,1\right)\text{,}\\ P=\left(1\pm \eta \right)\times \text{EV}\;\text{Pead}\;\text{Power}\;\text{Demand}\\ 0\leq P\leq 2\times \text{Max}\;\text{Power}\;\text{Demand.} \end{array}$$

In the above equations, η is the deflation factor. Adopting a normal distribution means that EV users rarely charge when their battery is empty or full (due to anxiety factor), but rather when it is below a threshold. A limit is placed on the total EV power demand *P*, which is two times the maximum power demand.

Now the resource vector ${\vec{Y}}_{j}^{\left[i\right]}$ and environment matrix $E^{\left[i\right]}$ are constructed using the location, demand and quantity as described above. $c_{f1}$ represents the latitude, $c_{f2}$ represents the longitude, and $c_{f3}$ represents the power demand of EVs:

(Equation 6) $${\vec{Y}}_{j}^{\left[i\right]}=\left(c_{j1},c_{j2},c_{j3}\right),E^{\left[i\right]}=\left(\begin{array}{l} {\vec{Y}}_{1}^{\left[i\right]}\\ {\vec{Y}}_{2}^{\left[i\right]}\\ \vdots \\ {\vec{Y}}_{n}^{\left[i\right]} \end{array}\right)=\left(\begin{array}{l} c_{11},c_{12},c_{13}\\ c_{21},c_{22},c_{23}\\ \vdots \\ c_{n1},c_{n2},c_{n3} \end{array}\right)\text{.}$$

**Figure 8:**
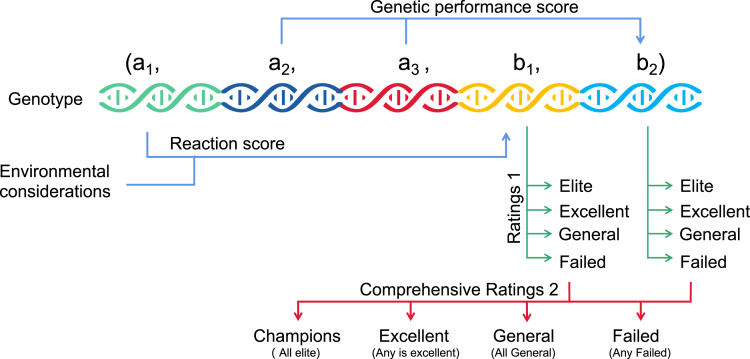
Example of a single gene in the genetic optimization The optimization process classifies a gene based on the target scores in the second half of gene vector ($b_{1},b_{2}$).

The shortest route of the EV to be charged, the total expenditures and the electrical load are considered in the optimization. The goal of the optimization is that the EV charging points be used for as many EVs as possible, the EV charging points work for as long as possible, and the least amount of total expenditures is invested to place the right type of charging points in the right locations. Therefore, these factors need to be optimized at the same time as a multi-objective optimization problem. This is shown in Figure 8.

#### Selecting the elite

An elite strategy is set up for each score through fuzzy logic as in Table 4. The multiple objective functions are integrated through such a combination of elite strategies to obtain a solution set for the optimization. The percentages in the Elite Strategy Rating depend on the specifics of the problem under study. The percentages are set heuristically and then are tuned based on the behavior of the gene vectors in the solution set of the optimization. In our case study, the bottom 20% are considered as unhealthy individuals that will be eliminated during the iterative process. The top 10% of individuals will generate 1 or 2 offspring individuals at random, 10–50% of individuals will only generate 1 offspring individuals, and 50–80% of individuals will generate 0 or 1 offspring individuals.

**Table 4:** Elite strategy rating

Rank	Elite	Excellent	General	Failed
Rating interval	[0%,10%)	(10%,50%)	(50%,80%)	(80%,100%)
Number of offspring	1 or 2	1	0 or 1	0

#### Utilization rate

The utilization rate is expressed in the following equation:

(Equation 7) $$\text{Utilization}\;\text{rate}=W_{1}\left(\frac{\text{Number}\;\text{of}\;\text{Feeded}\;\text{EVs}}{\text{Total}\;\text{Number}\;\text{of}\;\text{EVs}}\right)+W_{2}\left(1-\frac{1}{n}\sum_{i=1}^{n}\frac{{\left(b_{2}^{i}-T_{re}\right)}^{2}}{T_{re}^{2}}\right)\text{.}$$

The rate is formed as the weighted sum of two terms. The first term indicates the portion of EVs with their charging requested being satisfied. The working time of the charging points are expected to be around $T_{re}$. The quantity $T_{re}$ represents the recommended working time for the charging points, which is [10,15] hours.^43^ The second term shows the deviation of the charging point working hours from $T_{re}$. This term decreases by larger deviations. Here, $T_{re}=10$ hours is chosen with the weights $W_{1}=0.6,W_{2}=0.4$.

## Results and discussions

**Figure 9:**
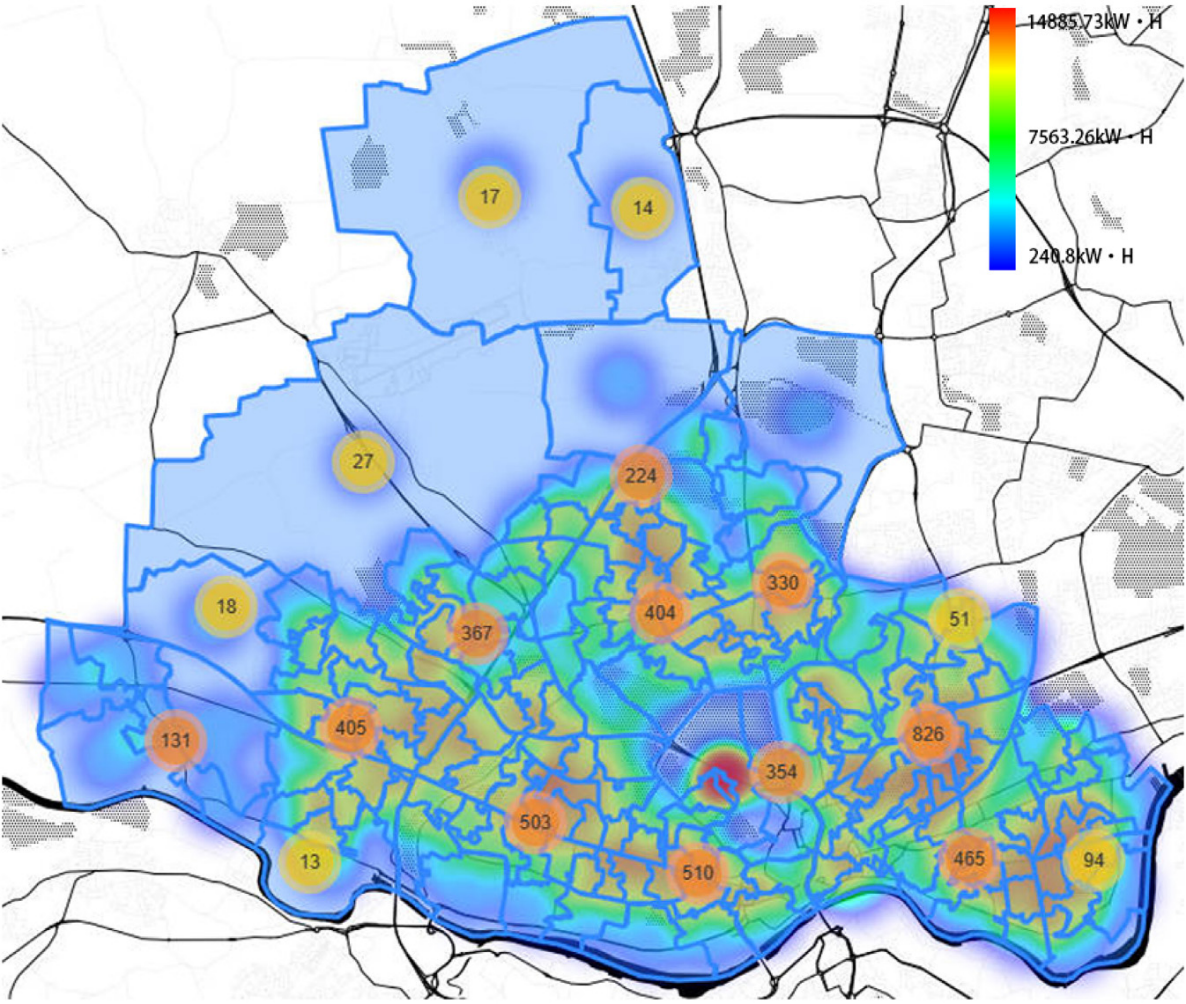
The cluster map of the results for Newcastle upon Tyne The numbers written on the circles represent the number of EV charging points within the related areas. This is related to *Leading the Way* scenario in peak year of 2042.

**Figure 10:**
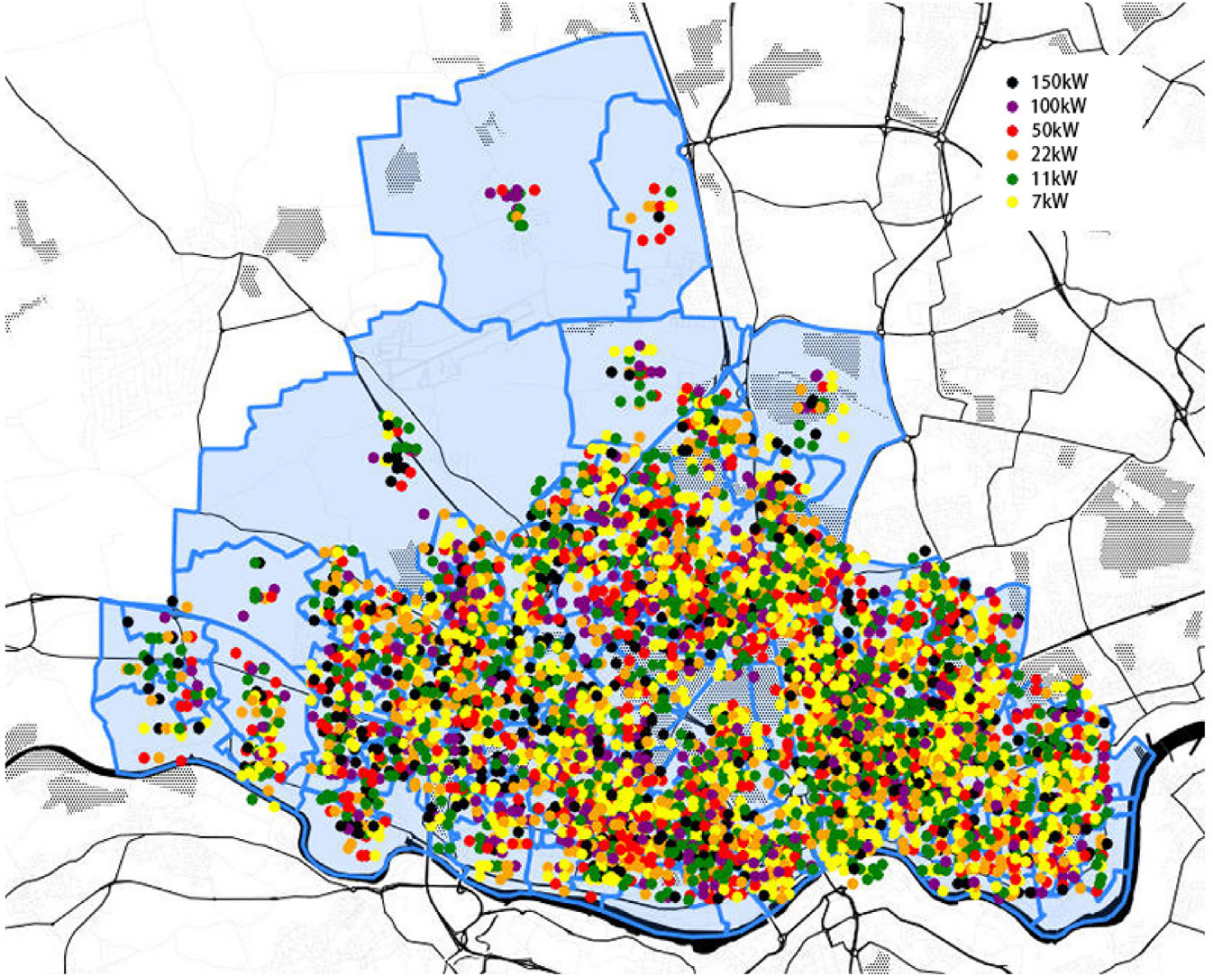
Spatial distribution of the genes in the optimization representing EV charging points The graph is related to the *Leading The Way* scenario peak year of 2042.

The optimization approach is applied for obtaining the charging locations in Newcastle upon Tyne considering the peak years of the four different scenarios reported in Table 3. Figure 9 shows the cluster map of the results for *Leading The Way scenario*. The numbers written on the circles represent the number of EV charging points within the related areas. The clustering is done by setting the map size to be 15,000 smaller than the actual size. The colors of the circles represent the proportion of the total number of charging points in the area to the total number of charging points (red color for higher portions). Figure 10 shows the spatial distribution of the genes in the optimization representing EV charging points. Color of the dots in the figure shows the type of the charging points. The optimization results indicate that by taking 4753 charging points and arranging them in the areas shown in Figure 10, a more appropriate efficiency of the charging points can be gained with lower total capital and operational expenditures. The two Figures 9 and 10 indicate that in rural areas where demand is low, fewer charging points are placed and close to the main roads. In urban areas, where there is a high demand, the number of charging points is high and they are located close to residential areas, shopping malls and major roads.

The relatively large number of charging points is computed by our optimization under the current and predicted road use behavior. In case policies are deployed that encourage a major shift to public transport and deter substantially the widespread private car usage, it is necessary to revise the scenarios, assumptions of the baseline model, and the predicted energy demand. This in turn affects the required number of charging points, but the overall spatial distribution of the charging points is expected to remain the same.

Table 5 gives the optimization results for the peak years of the four scenarios. The total number of charging points, average operating hours, average number of EV charging requests, and the total expenditures are reported. As can be seen from the table, the number of EVs are increasing from left to right for the four scenarios. The optimization algorithm also suggests that the required total number of charging points will increase with their total expenditures increasing as well. In contrast, the average operating hours of charging points is in the range [7.5,8], and goes up from 7.57 to 7.98 h. The average number of EV charging requests per charging point per day is almost a constant at 36 requests.

**Table 5:** Optimization results for peak years in different scenarios

Scenario	Leading The Way	Consumer Transformation	System Transformation	Steady Progression
Peak year	2042	2046	2048	2050
Quantity of EVs	134,606	145,345	146,617	160,403
Total number of charging points	4753	5167	5386	5817
Total Cost (£)	8,195,000	8,859,400	9,117,900	9,880,200
Average operating hours of charging points (h)	7.57	7.77	7.81	7.95
Average number of EV charging requests per charging point per day	35.79	36.17	35.39	35.36

**Figure 11:**
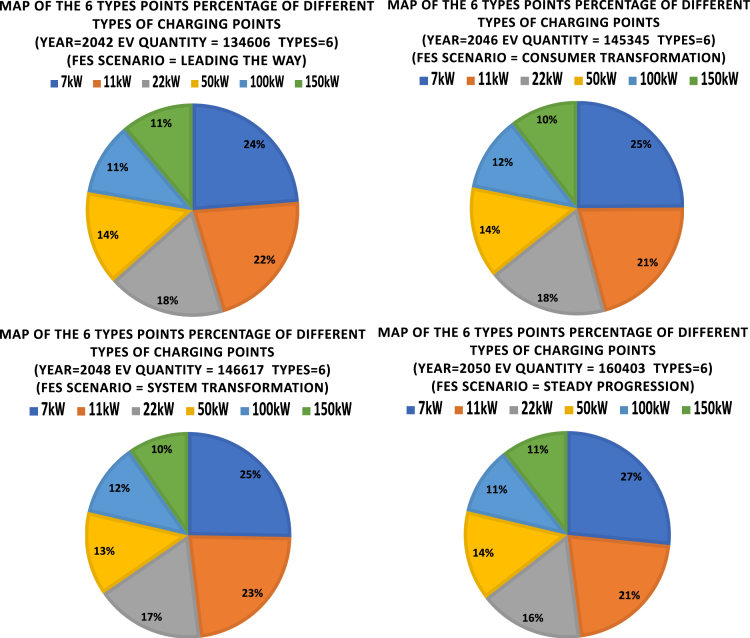
Portion of 6 types of EV charging points for the four scenarios The variations of the portions of different types are within 3% range of the total number of charging points. The optimal solution puts priority on the slower charging points.

Figure 11 shows the portion of 6 types of charging points for the four scenarios. The variations of the portions of different types are relatively small (within 3% range of the total number of charging points). It is found that regardless of the chosen initialization of the optimization process, the optimal solution puts priority on the slower charging points (respectively 7 kW, 11 kW, and 22 kW). The faster charging points (150 kW, 100 kW, and 50 kW) have smaller portions each around 10–13%. This means that while 7 kW slow charging dominates the market currently, it is more beneficial to improve charging efficiency and reduce investment costs with other types of charging points in the future charging point installations.

### Visualization of the iterative optimization

**Figure 12:**
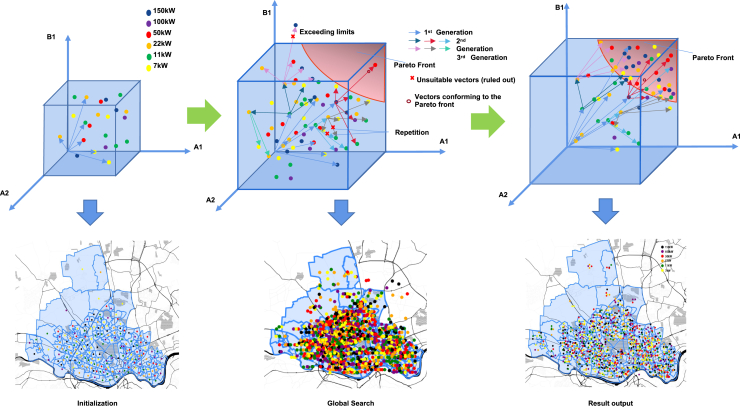
Optimization process of the multi-objective genetic algorithm The top figures show that the algorithm scores the available solutions by fuzzy logic and filters the better ones for the mutation-based generation of offspring to move them towards the optimal envelope. The bottom figures show the spatial visualization of the candidate charging points in the optimization process.

**Figure 13:**
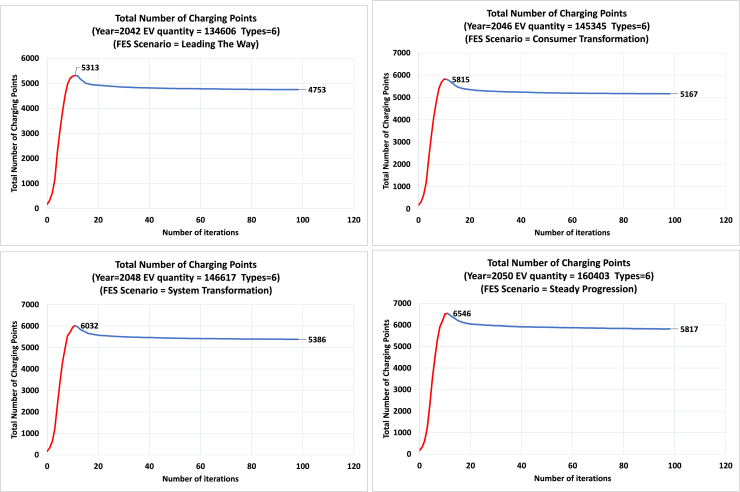
Total number of EV charging points in each iteration of the optimization Results for four different scenarios and 100 iterations. The total number of charging points starts from a very small value (initialization of the algorithm) and gradually increases to a peak value, then slightly decreases until converging to an optimal solution.

The optimization process is visualized in Figure 12. The algorithm initially places a number of charging points with different types in the center of the 175 areas. In the next step, the algorithm scores the available solutions by fuzzy logic and filters the better ones for the mutation-based generation of offspring. The generation of children in the next iteration of the algorithm expands the search direction and the search space. The first phase of the total iterations rapidly approximates the optimal envelope in the solution space. The second phase then combines different objectives through fuzzy logic to converge to an optimal solution. These two phases are also shown in Figure 13 that provides the total number of EV charging points in each iteration of the optimization. The first phase is indicated by the red color and the second phase by blue color. The results are for four different scenarios and 100 iterations. The total number of charging points starts from a very small value (initialization of the algorithm) and gradually increases to a peak value (first phase), then slightly decreases until converging to an optimal solution (second phase).

**Figure 14:**
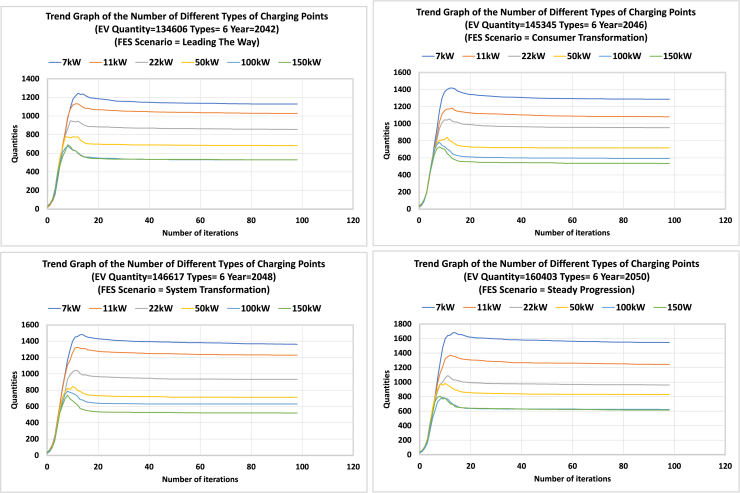
Number of 6 types of EV charging points in each iteration of the optimization Results for four different scenarios and 100 iterations. The required number of charging points starts from a small value (initialization of the algorithm) and gradually increases to a peak value, then slightly decreases until converging to an optimal solution.

**Figure 15:**
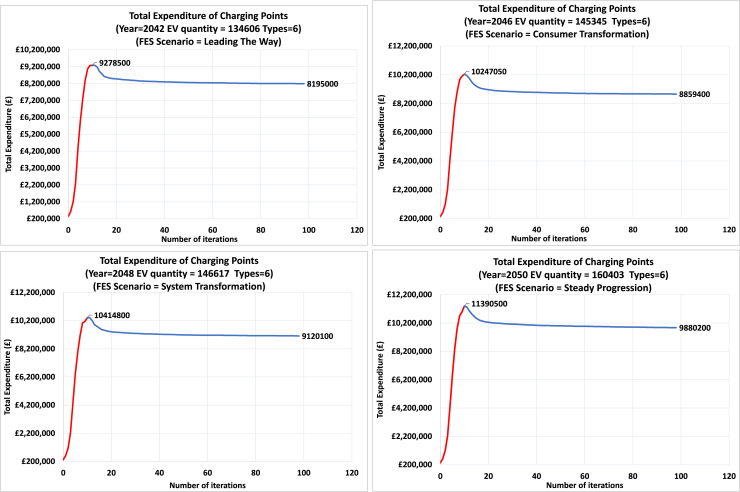
Total Expenditure of the EV charging points Results for four different scenarios and 100 iterations.

Figure 14 shows the number of 6 types of EV charging points in each iteration of the optimization. Results are for four different scenarios and 100 iterations. Similar to the total number of charging points, the number of each type starts from a small value and gradually increases to a peak value, then slightly decreases until converging to an optimal solution. A similar trend is observed in the total expenditure presented in Figure 15.

### Spatial distribution of the future charging infrastructure

**Figure 16:**
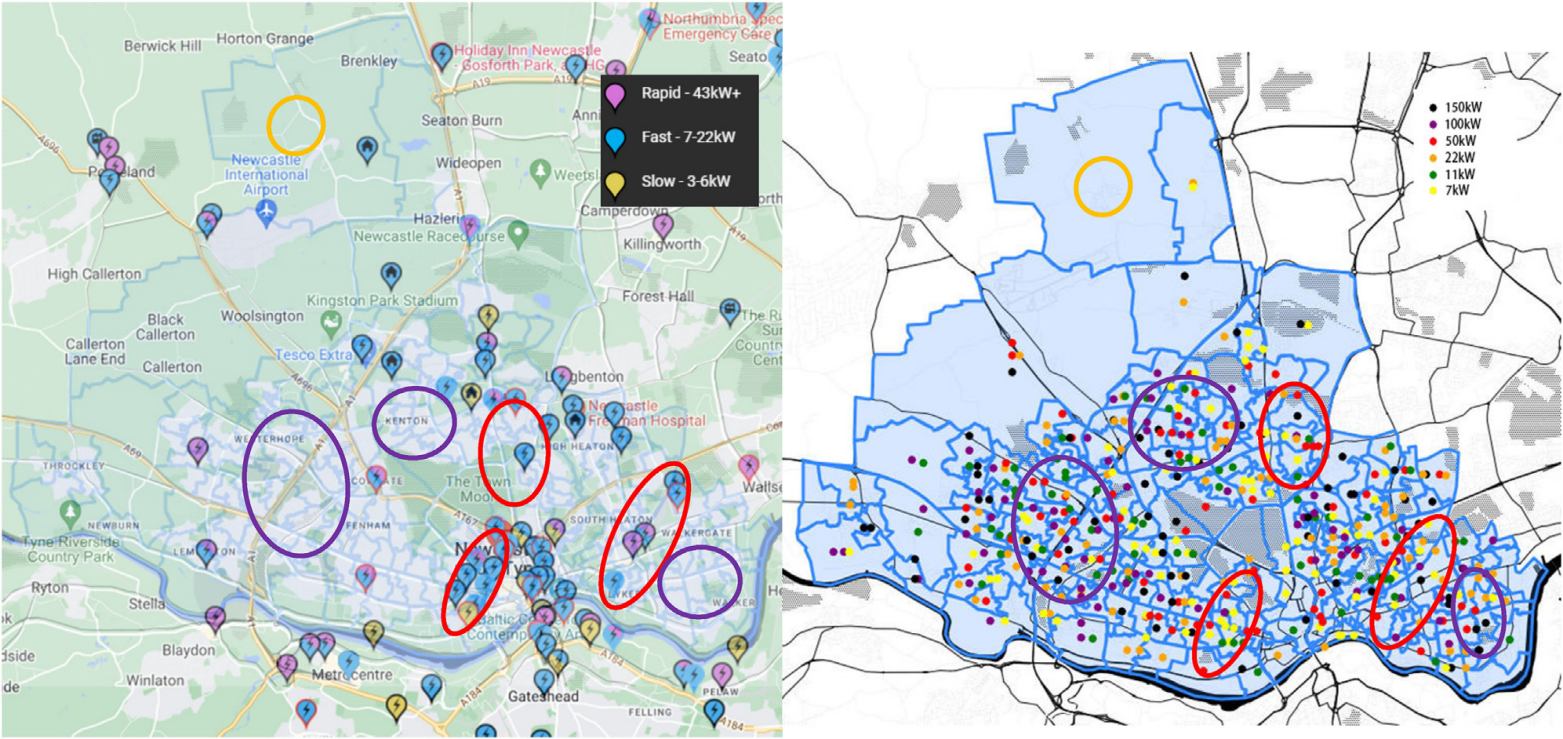
Spatial distribution of the charging points The left figure shows the existing charging points (source: ZAP-MAP). The right figure shows the solution of the optimization for future installations. The red ellipsoids represent similarities between the current charging points and the computed solution; The purple ellipsoids indicate areas with no installation but future installations are needed.

Figure 16 shows the current and computed spatial distribution of the charging points. The left figure shows the existing charging points (source: ZAP-MAP) and the right figure shows the solution of the optimization for future installations. The red ellipsoids represent similarities between the current charging points and the computed solution; The purple ellipsoids indicate areas with no installation but future installations is needed. This corresponds to residential areas that do not have a relevant arrangement of charging points at the moment.

### Validation

#### Sensitivity analysis of the optimization solution

Sensitivity analysis is an important validation technique to test the robustness of an optimization framework. In this study, sensitivity analysis has been conducted by altering input parameters, such as the number of EVs and the types of charging infrastructure. By analyzing the impact of these changes on the outcomes, it is demonstrated that the framework can adapt to different scenarios and produce meaningful results. This also helps to identify potential limitations and areas for improvement in the optimization method.

#### Sensitivity to the number of EVs

The sensitivity of the solution to the estimated number of EVs is studied by increasing and decreasing the EV numbers with 10%. The baseline for comparison is *Leading The Way* scenario for the year 2042, EV quantity 134606, and 6 types of EV charging points (cf. Table 3). The results are reported in Table 6.

**Table 6:** Sensitivity to the number of EVs

Changes in the EV numbers	+10%	−10%
Total number of charging points	+27.5%	−5.3%
Total expenditure (£)	+30.4%	−7.8%
Average number of EVs charged by a single charging point	−10.5%	+14.1%
Average operating hours of charging points (h)	−17.5%	+6.5%

Base of comparison is *Leading The Way* scenario for the year 2042, EV quantity 134606, and 6 types of EV charging points (cf. Table 3). The percentages are obtained by taking average over 6 runs of the optimization.

A 10% increase in the number of EVs to be charged increases the total number of charging points by 27.5%, increases the total expenditure by 30.4%, decreases the number of EVs to be charged by a single charging point by 10.5%, and reduces the average operating hours of a single charging point by 17.5%.

In contrast, a 10% reduction in the number of initial EVs would reduce the total number of charging points by 5.3%, reduce the total expenditure by 7.8%, increases the number of EVs to be charged by a single charging point by 14.1%, and increases the average operating hours of a single charging point by 6.5%.

#### Sensitivity to the number of charging types

Given the rapid developments in the charging point technologies, two different cases are considered to study the sensitivity of the optimization with respect to the diversity in the types of charging points. In the first case, it is assumed that only 5 types of charging points are available in the network by eliminating 150 kW charging points. In the second case, it is assumed that an additional type of 350 kW charging point is also available (in total 7 types).

The results are summarized in Table 7 by considering the base of comparison to be *Leading The Way* scenario for the year 2042 and EV quantity 134606 (cf. Table 3). The table shows that the number of charging points should be increased by 10.5% when charging points with higher powers are not available. However, smaller number of charging points are needed with high powers charging points available in the network. A small variation is also observed in the total expenditure, which is 3.36% more when larger number of charging points with smaller powers need to be installed. The changes in the number of charging points in turn influences the average number of EVs charged by a single charging point: smaller (larger) number of charging points will serve higher (lower) number of EVs in average when the demand is staying roughly the same (note that the network has a fixed number of EVs). Finally, the average operating hours of charging points has stayed almost the same. This means that the EV charging infrastructure should consider installing high power charging points when they become available and increase the diversity of the charging types.

**Table 7:** Sensitivity to the number of charging types

Number of charging point types	7 types	5 types
Total number of charging points	−7.87%	+10.5%
Total expenditure (£)	−0.85%	+3.36%
Average number of EVs charged by a single charging point	+7.39%	−9.43%
Average operating hours of charging points (h)	−1.03%	+0.24%

Base of comparison is *Leading The Way* scenario for the year 2042, EV quantity 134606, and 6 types of EV charging points (cf. Table 3). The percentages are obtained by taking average over 6 runs of the optimization.

**Figure 17:**
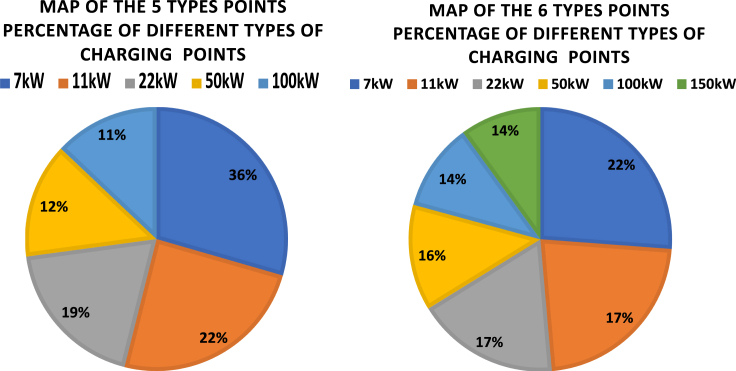
Portions of different types of charging points The left figure shows the results for five types of charging points (without 150 kW) and the right figure shows the results for 6 types.

Figure 17 shows the portions of different types of charging points when the network has only 5 types, and compares it with the 6 types. As can be seen in the left figure, 36% of charging points are 7 kW, but this is replaced in the right figure by 22% 7 kW and 14% 150 kW. Therefore, the optimization algorithm suggests that a portion of increase in the number of 7 kW charging points should be covered by installing high power 150 kW charging points.

### Testing the load carrying capacity

Load carrying capacity (LLC) is a concept used in various engineering disciplines to assess the reliability of system under extreme loads before any failure happening in the system.^44^^,^^45^ The goal of this section is to apply the LLC concept to the EV charging infrastructure and study its reliability under extreme conditions. For this purpose, the optimization of this paper is applied to design the EV charging infrastructure for the *Leading The Way* scenario for the year 2042 with EV quantity 134,606. The number of EVs is then increased to 300,000 (more than twice) and the response of the charging infrastructure to this extremely large demand is studied.

Table 8 shows the results of the LCC test. The number of EVs reported in the table is increased from 134,606 (Leading The Way Scenario) to 300,000 in order to capture an extreme situation. This means 123% more EVs are visiting Newcastle upon Tyne from other areas and need charging their EV batteries. Assuming that the total power demand will be twice as the normal power demand, the average operating hours of charging points will increase by 41%, and the average number of EVs charged by a single charging point is also increased by 76%. This means the designed EV charging infrastructure has the capability of absorbing the increased charging demand with the increased average operating hours.

**Table 8:** Testing the load carrying capacity for *Leading The Way* scenario when the charging infrastructure is designed for the year 2042 with EV quantity 134,606

Case	Normal	Extreme	Percentage of change
Number of EVs	134,606	300,000	123%
Number of charging point types	6 types	6 types	0%
Total number of charging points	4753	4753	0%
Total expenditure (£)	8,195,000	8,195,000	0%
Average number of EVs charged by a single charging point	35.79	63.11	76%
Average operating hours of charging points (h)	7.57	10.69	41%

### Comparing the new optimization with traditional genetic algorithm

Traditional GAs often encode characteristic parameters of the problem into binary (0 and 1) format, enabling the constructed gene vector to be recognized by machines and speeding up the computations. However, advancements in computer technology and the availability of various programming languages and libraries have eliminated the need for translating these parameters into binary format, simplifying problem expression and enhancing the use of vector space transformations.

When dealing with multi-objective optimization problems, traditional GAs typically compress and integrate multiple objectives into a single function, converting the problem into a single-objective optimization. This approach demands additional knowledge of the trade-offs between different objectives and necessitates multiple iterations of tuning the weights in the objective function. This process can be costly and time-consuming.

**Figure 18:**
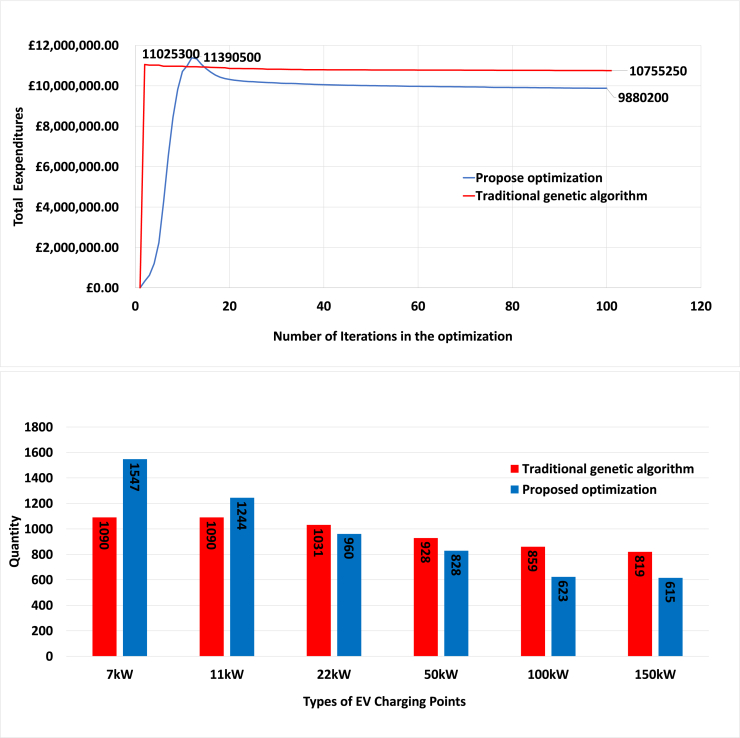
Comparing the new optimization method with the traditional genetic algorithm The results are for the year 2050, number of EVs 160403, 6 types of charging points, and steady progression scenario. The top figure shows the total expenditures as a function of iterations of the two algorithms. The bottom figure shows the quantities of the six charging types obtained by the traditional genetic algorithm and the proposed optimization.

In contrast, the optimization method proposed in this paper retains the multi-objective nature of the problem and provides a solution that automatically captures the trade-offs between the objectives. This is particularly advantageous when considering the distribution of total expenditures and quantities across different charging types. As Figure 18 (top) shows, the traditional GA suggests higher total expenditures for satisfying the EV energy demand. The quantities of six charging types obtained from the traditional GA and the proposed optimization is shown in Figure 18 (bottom). The approach of this paper suggests having higher number of slow and fast charging points, while traditional genetic optimization suggest a more uniform distribution for installing charging points.

By maintaining the multi-objective structure of the problem, the new optimization method offers a more comprehensive and efficient approach to balancing total expenditures and quantities across various charging types. This allows for more informed decision-making and resource allocation when designing and planning EV charging infrastructure, ultimately contributing to a more sustainable and accessible transportation ecosystem.

### Conclusions

This paper contributes to the AI-based support and assistance of implementing policy commitments by capturing the focus of the implementation problem, and building a framework that integrates data with simulation and optimization. It also provides new ideas and solutions for automatic intelligent decision-making. With reference to deep learning neural networks and GAs, the approach of this paper created computational models capable of simultaneous global search and unsupervised learning. The model is proven to understand statistical data and automatically generate acceptable, intelligent decisions with good robustness.

This paper built a model for simulating the net zero emission policy commitment on EV charging infrastructure to compute the increase in the quantity of charging points with different types. The model includes two distinctive stages of simulation and optimization. The simulation stage of the baseline model has been taken from the industrial partner, Arup Group Limited. The data of traveled distances were utilized and the model was customized to simulate the interaction between EV users and charging points within the model. The main contribution of the results presented in this paper was on the development of a novel optimization approach to find the optimal location of EV charging points (best implementation of the policy commitment) utilizing the baseline simulation model. The relevant subsets of the output of the simulation model is used for feeding the optimization stage of the model.

With focus on the EV charging infrastructure of Newcastle upon Tyne, UK, the optimization model is used to estimate and optimize the charging points types, charging points quantity, charging points locations, total expenditures, and utilization of charging points. The optimization was performed for four different future energy scenarios and the demand and EV quantities of the peak years for Newcastle upon Tyne were used. Quantitatively, the variations of the portions of different types of charging points for the four scenarios are relatively small and within 3% range of the total number of charging points. The optimal solutions put priority on the slower charging points, with faster charging points having smaller portions each around 10–13%. The optimization shows that while 7 kW slow charging dominates the market currently, it is more beneficial to improve charging efficiency and reduce investment costs with other types of charging points in the future installations. Moreover, in the leading scenario for the year 2042 with 134,606 EVs, a total of 4,753 charging points is recommended, resulting in a total cost of £8.2 million and an average operating time of 7.57 h per charging point. The results also illustrate the spatial distribution of charging points, with higher concentrations in urban areas and near major roads.

In order to study the factors influencing the outcome of the optimization, a sensitivity analysis was carried out on the number of EVs and the type of charging points. The optimization results confirm that having more diversity in the charging point types and installing more high-power points can reduce the total expenditure while having similar performance in addressing the charging demand. The analysis reveals the adaptability of the optimization framework to different scenarios, providing valuable insights for decision-making and highlighting potential areas for improvement.

The optimization approach proposed in this paper is general and can be applied to any baseline model that provides estimates of the future EV charging demands in any specific area. In the future, the multi-objective optimization framework will provide a more scientific and comprehensive support for the EV charging infrastructure in the context of net zero emission strategy.

Future research will focus on further enhancing the simulation model by incorporating additional factors such as demographic and socioeconomic variables. Additionally, advanced optimization techniques can be explored to address the computational complexities and improve the efficiency of the optimization process. These advancements will contribute to the ongoing efforts in developing intelligent and adaptive solutions for EV charging infrastructure planning.

### Strengths and weaknesses

The GA optimization model allows complex problems to be programmed in a simplified way by constructing search and environment matrices, gene vectors and resource vectors, and then updating these vectors and matrices in an iterative process. It involves employing operations such as encoding, non-dominated sorting, decoding, and multi-objective compression of single objectives. The optimization approach of this paper can be applied to any baseline model that generates the behavior of EV charging demands within any spatial boundaries and be integrated with real datasets.

However, the optimization approach of this paper comes with its own limitations. The accuracy and reliability of obtained solution depends on the underlying model that generates the EV demands and also on the quality of the datasets used. The operation of constructing gene vectors using multiple parameters and thus improving accuracy and reliability increases the dimension of the vectors and demands higher computational power. Constructing multidimensional vectors for optimization also generates complex score calculations, which in turn increases the required computational time. To address the computational complexity, it is crucial to take the appropriate parameters and the main influencing factors for constructing the optimal envelope using the optimization approach.

## STAR★Methods

### Key resources table

**Table:** 

REAGENT or RESOURCE	SOURCE	IDENTIFIER
**Deposited data**		

Car Ownership	UK Department For Transport	https://www.data.gov.uk/dataset/11bc7aaf-ddf6-4133-a91d-84e6f20a663e/national-trip-end-model-ntem
Vehicle Availability	UK Office for National Statistics (Nomis)	https://www.nomisweb.co.uk/home/Search?context=&term=Car+or+van+availability
Power Profile	UK Department For Transport	https://www.gov.uk/government/statistics/electric-chargepoint-analysis-2017-domestics
Trip Statistics	UK Office for National Statistics (Census)	https://www.ons.gov.uk/census/2011census/2011censusdata/originanddestinationdata
EV Efficiency and EV Battery Size	Bloomberg	https://www.bloomberg.com/professional/datasets/?bbgsum-page=DG-WS-PROF-SOLU-DATACONT&mpam-page=21140&tactic-page=429341
Future Energy Scenarios	National Grid	https://www.nationalgrideso.com/future-energy/future-energy-scenarios

**Software and algorithms**		

Python 3.10	Python Software Foundation	https://www.python.org
Code for model building and evaluation	Github repository	https://github.com/farzanehfar/MultiObjective-Optimization

### Resource availability

#### Lead contact

Further information and requests for resources should be directed to and will be fulfilled by the lead contact, Farzaneh Farhadi (F.Farhadi2@newcastle.ac.uk)

#### Materials availability

This study did not generate new unique materials.

### Methods details

#### Multi-Objective Genetic Algorithm Optimization Inspired by LSTM

First build the set of gene vectors:

(Equation 8) $${\vec{X}}_{j}^{\left[i\right]}=\left(a_{j1},a_{j2},\ldots ,a_{jn},b_{j1},b_{j2},\ldots ,b_{jn}\right),j=1,2,\ldots ,m\text{.}$$

In the above notation of ${\vec{X}}_{j}^{\left[i\right]}$, j represents different genes within the same generation, and [i] represents the generation number (or iteration number). The first half of the gene vector $\left(a_{j1},a_{j2},\ldots ,a_{jn}\right)$ represents the characteristics of the gene itself, including *coordinates*, *size*, *length*, and *weight*. This part will be called the sequence of characteristics. The second half of the gene vector $\left(b_{j1},b_{j2},\cdots ,b_{jn}\right)$ represents the expression shape of the characteristic sequence after filtering by a specific environment or scenario. This part will be called the *target score sequence* (or expression sequence). Their relationship will be discussed later in this section.

Inspired by the Long Short-Term Memory (LSTM) models in Machine Learning for vector processing, the gene vectors are included into a matrix to form the space of the solution set V:

(Equation 9) $$V^{\left[i\right]}=\left(\begin{array}{l} {\vec{X}}_{1}^{\left[i\right]}\\ {\vec{X}}_{2}^{\left[i\right]}\\ \vdots \\ {\vec{X}}_{m}^{\left[i\right]} \end{array}\right)=\left(\begin{array}{l} a_{11},a_{12},\ldots ,a_{1n},b_{11},b_{12},\ldots ,b_{1n}\\ a_{21},a_{22},\ldots ,a_{2n},b_{21},b_{22},\ldots ,b_{2n}\\ \vdots \\ a_{m1},a_{m2},\ldots ,a_{mn},b_{m1},b_{m2},\ldots ,b_{mn} \end{array}\right)\text{.}$$

The vector ${\vec{Y}}_{j}$ is also constructed to characterise the features to be solved. This vector represents the feature expression. This will be called the *resource vector*:

(Equation 10) $${\vec{Y}}_{j}^{\left[i\right]}=\left(c_{j1},c_{j2},\ldots ,c_{jn}\right),j=1,2,\ldots ,m\text{.}$$

Similarly, the resource vectors are included into a matrix to construct the environment space matrix. The environment matrix maps actual data from external sources to simulate a realistic environment.

(Equation 11) $$E^{\left[i\right]}=\left(\begin{array}{l} {\vec{Y}}_{1}^{\left[i\right]}\\ {\vec{Y}}_{2}^{\left[i\right]}\\ \vdots \\ {\vec{Y}}_{m}^{\left[i\right]} \end{array}\right)=\left(\begin{array}{l} c_{11},c_{12},\ldots ,c_{1n}\\ c_{21},c_{22},\ldots ,c_{2n}\\ \vdots \\ c_{m1},c_{m2},\ldots ,c_{mn} \end{array}\right)\text{.}$$

By calculating the gene vector and the resource vector, the performance scores of the gene vector can be obtained and recorded in the second half of the gene vector. The recorded scores are multiple objective functions of the genetic algorithm. Scores that require a response to the environment are reaction scores. Scores that require recording the performance of internal gene combinations are genetic performance scores. In general, this can be denoted as

(Equation 12) $$b_{jk}^{\left[i\right]}=F_{jk}\left(a_{j1},a_{j2},\ldots ,a_{jn},c_{j1},c_{j2},\ldots ,c_{jn}\right),\text{for}\;\text{all}\;j,k\text{.}$$

which shows that in each generation [i], $b_{jk}$ is a function of the characteristics of the gene and the resource vector.

Then, the gene vectors are filtered according to their target scores $b_{jk}$:

(Equation 13) $$V^{\left[i\right]}=\left\{{\vec{X}}_{j}^{\left[i\right]}\;|\;l_{1}<b_{jk}<l_{2},b_{jk}\in X_{j}^{\left[i\right]},j=1,2,\ldots ,n\right\}\text{,}$$

where $l_{1}$ and $l_{2}$ represent the lower and upper limits. The genotype is considered to be environmentally compatible when the value of $b_{jk}$ is within the range $\left(l_{1},l_{2}\right)$. The rest of the vectors in the solution vector space are considered as not required and are eliminated. *Fuzzy Logic* is used to assign a performance score to each gene vector and guide the search toward the optimal envelope.

Fuzzy Logic Elite Strategy

**Table:** 

Elite	Excellent	General	Failed
[0,a]	(a,b]	(b,c]	(c,d]

As shown in Figure 8, the fuzzy logic ratings are used for every target score in the solution vector to analyze whether the genes have met the requirements. The above table is used for this classification. A second rating of the entire vector score is performed to identify the elite and failed genes.

The first rated elite is the one that performs well in a specific objective score, which means that the vectorisation direction is likely to be the optimal solution direction for a single objective. With the first rating, the offspring produced by elite is controlled and thus possible solutions in that direction is searched. The second composed rating enables a global view to be taken to rate the exemplar genes in each generation who has performed very well in all of the multi-objective scores and who is likely to be the direction of the optimal solution for the multi-objective optimization. In the second composed rating, a gene that is failed in any target score will be recognised as failed. However, every time a second composed rating is only a part of the performance of the data mapping and cannot be generalised across the entire data, these exemplar genes will be memorised in the generation iterations to ensure that the optimised result cover the full dataset.

Both crowding and search direction are controlled by comparing the cosine similarity of gene vectors to the exemplar vectors in each generation:

(Equation 14) $$Elite=\left\{X^{\left[i\right]}\;|\;X^{\left[i\right]}>R\left(a,b,c,d\right)\right\}\text{,}$$

where R(a,b,c,d) represents the rating criteria, and

(Equation 15) $$\text{cosine}\left(X_{e},X_{j}^{\left[i\right]}\right)=\frac{a_{e1}a_{j1}^{\left[i\right]}+a_{e2}a_{j2}^{\left[i\right]}+\ldots +b_{en}b_{jn}^{\left[i\right]}}{\sqrt{a_{e1}^{2}+a_{e2}^{2}+\ldots +b_{em}^{2}}\cdot \sqrt{a_{j1}^{\left[i\right]2}+a_{j2}^{\left[i\right]2}+\ldots +b_{jn}^{\left[i\right]2}}}\text{.}$$

If the cosine similarity is large, the environment has not changed significantly. The algorithm can perceive the changing trends in the environment by recording exemplar vectors in each generation. The rating criteria for fuzzy logic are computed by combining the scores of the exemplar set and of new ones:

(Equation 16) $$R\left(a,b,c,d\right):=\beta R{\left(a,b,c,d\right)}_{\text{champions}}+\left(1-\beta \right)R{\left(a,b,c,d\right)}^{\left[i\right]}\text{.}$$

This is similar to unsupervised learning with β being the learning Rate.^46^ The algorithm learns to recognise good and bad decisions in multiple iterations of processing data and making decisions.

The LSTM model constructs memory gates, forget gates, and output gates through the *sigmoid* function and *tanh* function, thus remembering important data and forgetting the unimportant ones.^46^ The previously processed information is then passed on to the next nerve cell. This structure is adapted for the new optimization of this paper based on the genetic algorithm. In the initialisation, arbitrary decision scenarios are designed to react to the mapping environment. The response is then scored in gene vectors. This process generates a certain number of offsprings. Then the regularised offspring is added to $V^{\left[i\right]}$ to form the revised matrix $V^{\left[i+1\right]}$.

In order to produce offspring in the next iteration and move toward the better optimal solutions, the vector space matrix is transformed randomly with some spatial directionality to expand the more prominent search space. Therefore, the random transformation vector is constructed to achieve crossover and mutation manipulation of genotypes:

(Equation 17) $${\vec{T}}_{j}^{\left[i\right]}=\left(t_{j1},t_{j2},\ldots ,t_{jn}\right)\text{.}$$

Each gene vector is manipulated using tensor multiplication to generate new offspring:

(Equation 18) $${\vec{X}}_{j}^{\left[i+1\right]}={\vec{T}}_{j}^{\left[i\right]}⊗{\vec{X}}_{j}^{\left[i\right]}=\left(t_{j1}a_{j1},t_{j2}a_{j2},\cdots ,t_{jn}a_{jn}\right)\text{.}$$

The transformed vectors are then added to the solution vector space, thus forming a new solution vector space:

(Equation 19) $$V^{\left[i+1\right]}=\left(\begin{array}{l} {\vec{X}}_{1}^{\left[i\right]}\\ {\vec{X}}_{2}^{\left[i\right]}\\ \vdots \\ {\vec{X}}_{m}^{\left[i\right]}\\ {\vec{X}}_{1}^{\left[i+1\right]}\\ {\vec{X}}_{2}^{\left[i+1\right]}\\ \vdots \\ {\vec{X}}_{m}^{\left[i+1\right]} \end{array}\right)\text{.}$$

In each iteration, the responses of the previous generation are compared with the new environment, and a score is calculated. The scores are used to generate a matrix of children to perform the search, thus ensuring that global search and unsupervised learning are mutually reinforcing. These steps are designed specifically to provide a model of continuous learning from data and experience.

## Data Availability

• Original datasets for electric vehicle charging optimization are publicly available with links listed in the key resources table. • All original code has been deposited at Github and is publicly available as of the date of publication. DOI is listed in the key resources table. • Any additional information required to reanalyse the data reported in this paper is available from the lead contact upon request.
